# Integrated circadian regulation in horticultural plants: light-environment mechanisms governing growth and development

**DOI:** 10.1093/hr/uhag056

**Published:** 2026-02-23

**Authors:** Zhi-Hang Hu, Nan Zhang, Ting Huang, Chen Chen, Jing Zhuang, Ai-Sheng Xiong

**Affiliations:** College of Horticulture, Nanjing Agricultural University, Nanjing 210095, China; State Key Laboratory of Crop Genetics & Germplasm Enhancement and Utilization, Nanjing Agricultural University, Nanjing 210095, China; College of Horticulture, Nanjing Agricultural University, Nanjing 210095, China; State Key Laboratory of Crop Genetics & Germplasm Enhancement and Utilization, Nanjing Agricultural University, Nanjing 210095, China; College of Horticulture, Nanjing Agricultural University, Nanjing 210095, China; College of Sciences, Nanjing Agricultural University, Nanjing 210095, China; College of Horticulture, Nanjing Agricultural University, Nanjing 210095, China; State Key Laboratory of Crop Genetics & Germplasm Enhancement and Utilization, Nanjing Agricultural University, Nanjing 210095, China; College of Horticulture, Nanjing Agricultural University, Nanjing 210095, China; College of Horticulture, Nanjing Agricultural University, Nanjing 210095, China; State Key Laboratory of Crop Genetics & Germplasm Enhancement and Utilization, Nanjing Agricultural University, Nanjing 210095, China

## Abstract

The circadian clock enables plants to synchronize physiological and developmental processes with daily and seasonal light fluctuations. In horticultural crops, this endogenous oscillator interacts with photoperiod, light quality, and light intensity to coordinate flowering, growth, metabolism, and stress adaptation. Photoperiodic control, mediated largely by the conserved CONSTANS (CO)–FLOWERING LOCUS T (FT) module, governs flowering transitions and vegetative–reproductive balance in horticultural crops, such as strawberry, chrysanthemum, cucumber, tomato, and potato. Spectral composition, particularly red/far-red and blue light perceived through phytochromes and cryptochromes, reshapes circadian amplitude and phase to regulate photosynthesis, morphogenesis, and secondary metabolism. Meanwhile, light intensity adjusts oscillator robustness and energy allocation, influencing rhythmic stability under controlled-environment cultivation. The emerging research topics such as on species-specific clock diversity, circadian regulation of quality traits, and precision lighting strategies aligned with rhythmic principles were also discussed. Analyzing the interaction between light signals and the biological clock will help deepen our understanding of the time regulation mechanism in horticulture plants, and can provide a basis for designing optimized periodic cultivation systems in horticulture, thereby improving yield and quality of horticultural crops. In this review, we will summarize the research findings on how light environments regulate the circadian rhythms of horticultural plants, as well as their potential applications in horticulture.

## Introduction

The higher plant circadian clock was a fundamental endogenous timing mechanism that orchestrated internal physiological and developmental processes in accordance with diurnal and seasonal environmental rhythms [1, 2]. Constructed of interlocked transcription–translation feedback loops, it generated self-sustained oscillations of approximately 24 h even in the absence of external zeitgebers. Through entrainment by light and temperature cues, the clock ensured temporal coordination between endogenous rhythms and environmental cycles [2]. In horticultural plants, this system was vital for adapting to latitudinal photoperiodic variation, seasonal changes, and artificial lighting regimes, thereby exerting profound influence on growth, development, and productivity of horticultural plants [3, 4].

Research on higher plant circadian rhythms dates back to the early 20th century. Garner and Allard first discovered the photoperiodic phenomenon in tobacco and soybean, proposing that plants perceived day length to regulate flowering and other developmental processes [5]. This pioneering work established the foundation for studying photoperiodic control of flowering and revealed that plants possess an internal timing mechanism to measure day and night length [5]. Numerous subsequent studies demonstrated that higher plants adjusted metabolism, photosynthesis, hormone levels, and stress responses according to diurnal cycles. Sunflower (*Helianthus annuus*) tracked the sun from east to west and reoriented toward the east at dawn [6]. *Arabidopsis thaliana* precisely degraded starch during the night to sustain metabolism until morning [7]. Many ornamental species bloomed or emitted fragrance at specific times of day, forming the classical ‘Linnaeus flower clock’. These phenomena underscored the crucial role of the circadian clock in enabling higher plants to adapt to environmental rhythms [8, 9].

Light served as an important external cue (zeitgeber) for circadian entrainment. Clock synchronization was also influenced by other signals, including temperature changes, metabolic conditions, and carbon content, and may vary among different tissues. Light encompassed multiple dimensions—photoperiod (day length), light quality (spectral composition), and light intensity [10, 11]. In higher plants, light signals were perceived through various photoreceptors, including phytochromes (red/far-red light receptors) and cryptochromes (blue light receptors), which modulated the transcription of core clock genes and thereby regulated downstream developmental processes [12, 13]. Beyond daily light–dark transitions, seasonal variations in day length provided predictable environmental information. Many higher plant species had evolved mechanisms to interpret photoperiodic cues, allowing them to initiate flowering, fruiting, or dormancy at appropriate times of the year [1, 2]. Elucidating the interactive mechanisms between the circadian clock and the light environment was fundamental to plant biology and offered valuable guidance for the cultivation management and breeding of horticultural crops.

Horticultural crops, as an important group of plants closely related to human daily life, have attracted increasing attention in recent years. Research in this field has also gradually expanded from model species, such as *Arabidopsis* to horticultural crops. In the past few years, advances in horticultural crops research have made rapid progress. The horticultural crops mainly included strawberry, tomato, cucumber, chrysanthemum, tea plant, celery, carrot, grape, and other woody perennial horticultural plants. These horticultural crops cover major types of fruit, vegetable, flower, tea plants, and medicinal plants. Research on the biological clock, circadian rhythm, and the preliminary mathematical model have also linked the regulation of circadian with related traits of production in horticultural crops. The interaction between the circadian rhythms of horticultural crops and the light environment was dynamic and bidirectional. Light environment continuously regulated the phase, period, and amplitude of the biological clock; and the biological clock controlled light perception and downstream signal transduction in a time-dependent manner. The same light environment may trigger different molecular and physiological responses at different internal biological clock stages of horticultural crops.

This review summarized the latest advances in circadian clock research in horticultural plants, integrating knowledge from model species, such as *Arabidopsis* and *Oryza sativa*, to elucidate the core mechanisms. We outlined the central oscillatory network and major discoveries in model systems, which served as a theoretical basis for understanding circadian regulation in horticultural plants. Then, we focused on the responses of horticultural plant clocks to three major light-related factors: photoperiod, light quality, and light intensity. The section on photoperiod examined physiological and molecular responses to changes in day length, including photoperiodic flowering induction and the transition between vegetative and reproductive growth. The section on light quality explored how spectral components, particularly red, far-red, and blue light affected circadian regulation and developmental outputs through specific photoreceptors. The section on light intensity addressed how irradiance modulated circadian amplitude and plant development. We also outlined the mathematical models used for understanding of the core components and regulatory networks of the plant circadian clock. Finally, we discussed emerging research priorities and challenges, identified knowledge gaps, and outlined future perspectives for circadian research in horticultural crops.

## Core mechanisms of the circadian clock in higher plants

**Figure 1:**
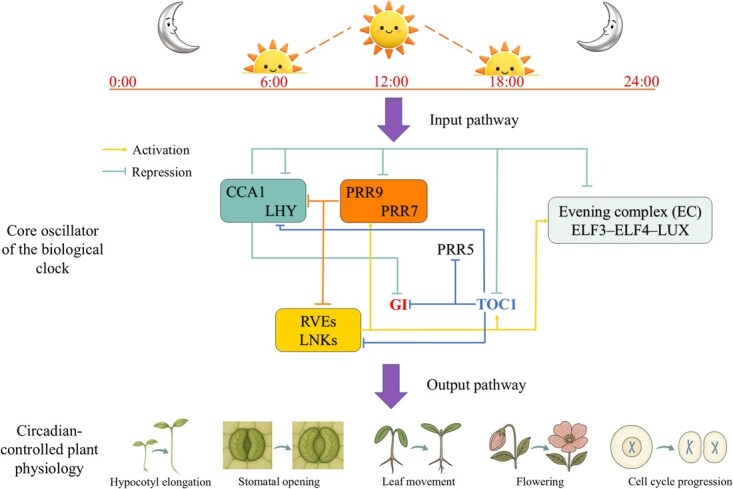
Schematic model of the higher plant circadian clock and its regulation of physiological process. The diagram illustrated the molecular network of the higher plant circadian clock, including its core oscillator, input, and output pathways. A blunt solid line at the terminal end represented genes exerting inhibitory effects in a negative feedback loop. The arrow indicated the genes that had an activating role in the regulatory network. Downstream output pathways regulated circadian-controlled physiological processes, including hypocotyl elongation, stomatal opening, leaf movement, flowering, and cell cycle progression. Sun and moon icons at the top represented diurnal and nocturnal phases over a 24-hour period (0:00–24:00).

In *Arabidopsis*, the core circadian clock consisted of a series of interlocked transcription–translation feedback loops comprising morning, daytime, and evening phased genes (Fig. 1) [14–17]. At dawn, the morning-phased genes encoding transcription factors CIRCADIAN CLOCK ASSOCIATED 1 (CCA1) and LATE ELONGATED HYPOCOTYL (LHY), which belong to the MYB family proteins, were highly expressed and subsequently inhibited the transcription of themselves and other phase-specific genes [14, 18]. As CCA1 and LHY proteins gradually degraded during the day, a group of PSEUDO-RESPONSE REGULATORS (PRRs), including PRR9, PRR7, PRR5, and the evening-expressed PRR1/TOC1, were sequentially activated. These PRR proteins acted as transcriptional repressors, peaking from afternoon to dusk to inhibit the re-expression of morning genes, such as *CCA1* and *LHY* [19]. During the evening, another set of genes known as the ‘Evening Complex’ (EC) comprising *LUX ARRHYTHMO* (*LUX*), *EARLY FLOWERING 3* (*ELF3*), and *EARLY FLOWERING 4* (*ELF4*) was expressed after sunset. These components formed a repressive complex that inhibited the transcription of *TOC1* and other PRR genes at night [20]. Meanwhile, morning-phase activators, such as REVEILLE8 (RVE8), another clock-controlled MYB factor, promoted the afternoon activation of EC components [15]. Through these multilayered feedback loops of repression and activation, the clock network ensured that each gene group was expressed at the appropriate time of day, generating a robust 24-hour oscillation. In *Arabidopsis*, several additional regulators enhanced the stability and flexibility of the circadian system. For instance, GIGANTEA (GI) and LNK1/LNK2 connected the clock to light signaling pathways [21, 22], while photo-regulated F-box proteins, such as ZEITLUPE (ZTL) and FLAVIN-BINDING KELCH REPEAT F-BOX1 (FKF1), transmitted photic cues to clock components by modulating protein stability [12, 13]. These interactions enabled the clock to maintain temporal precision under constant conditions while retaining plasticity in response to environmental changes, such as light and temperature fluctuations. This dynamic balance between stability and flexibility was considered a key evolutionary advantage for plant adaptation.

Another important plant, rice (*O. sativa*), possessed homologous clock genes in its genome. These clock genes had specific functions in rice that differed from those in *Arabidopsis*. In rice, *heading date 1* (*Hd1*) [23], a CONSTANS ortholog, functioned as a key photoperiod regulator by promoting heading under short days but repressing flowering under long days through modulation of florigen expression (*Hd3a*/*RFT1*). Conversely, in *Arabidopsis*, CO activates *FT* under long days to induce flowering [24]. The core circadian rhythm structure of angiosperms exhibits a high degree of conservation. During the evolution of plant, the functions of specific components had undergone various changes. Functional characterizations of clock genes in model plants provided valuable insights into the evolutionary plasticity and environmental adaptability of circadian systems in horticultural plants [25]. Comparative researches indicated that circadian systems diversified *via* duplication and functional divergence of clock and light/temperature input modules, together with regulatory rewiring of downstream outputs. This evolutionary plasticity and natural variation could tune period, phase, and amplitude to improve environmental adaptability across horticultural plants occupying distinct photoperiodic and thermal niches [26, 27].

**Figure 2:**
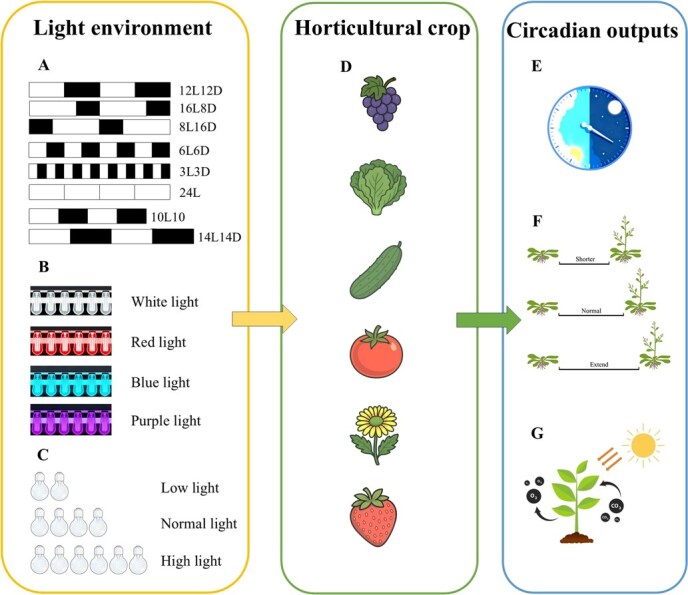
Effects of photoperiod, light quality, and light intensity on circadian regulation and growth of horticultural plants. (A) Representative photoperiod regimes commonly used in horticultural research, including standard light–dark cycles (12L12D, 16L8D, 8L16D) [32], skeleton photoperiods (6L6D, 3L3D) [33], continuous light (24 L) [33], and extended or shortened daylength treatments (10L10D, 14L14D) [34]. (B) Different light qualities used to investigate spectral regulation of circadian rhythms and developmental processes. (C) Light intensity gradients (low, normal, and high light) representing the quantitative aspect of irradiance effects on photosynthesis and diel rhythmicity. (D) Representative horticultural species-grape, lettuce, cucumber, tomato, chrysanthemum, and strawberry-used as crops in circadian and light-environment researches. (E) Illustration of the circadian clock highlighting phases driven by day–night transitions. (F) Photoperiod-dependent morphological responses, including shortened, normal, or extended growth patterns under different light regimes. (G) Overall physiological outputs regulated by circadian–light interactions, including photosynthesis, carbon assimilation, and metabolic homeostasis.

Overall, researches in model plants (*Arabidopsis*) established a general framework for understanding plant circadian organization: the core oscillator-maintained rhythmicity through self-sustained transcriptional feedback loops; input pathways entrained the oscillator to light–dark and temperature cycles; and output pathways transmitted timing information to regulate downstream physiological and developmental processes [28, 29]. The biological clock was capable of coordinating the growth and development process, enhancing the tolerance to both biotic and abiotic stresses [30, 31]. The following sections built upon this theoretical foundation to explore how the circadian clock interacted with photoperiod, light quality, and light intensity in horticultural plants (Fig. 2).

## Circadian regulation of photoperiodic responses in horticultural plants

Photoperiod referred to the relative duration of light and darkness within a 24-hour period. Many horticultural plants were highly sensitive to changes in day length and regulated key developmental transitions, such as flowering, tuberization, and vegetative growth through circadian-controlled photoperiodic responses [35, 36]. Based on their flowering behavior, higher plants were generally classified into short-day plants (SDPs), long-day plants (LDPs), and day-neutral plants (DNPs) [37, 38]. This classification had direct implications for horticultural crops management and breeding. For example, chrysanthemum, strawberry, and potato were typical SDPs, whereas spinach and lettuce were LDPs; tomato and cucumber were generally considered DNPs, and some wild or local cultivars retained photoperiod sensitivity.

### Photoperiodic regulation of flowering in horticultural plants

Photoperiodic induction of flowering was a crucial process in the transition from vegetative to reproductive growth. The circadian clock functioned as both a ‘timer’ and a ‘coordinator’, integrating rhythmic gene expression with environmental light cues to determine day length [39]. In *Arabidopsis*, the external coincidence model proposed that the expression of CO gene peaked in the evening, and flowering occurred when CO gene expression coincided with light exposure under long-day conditions. Stabilized CO protein then activated *FT* gene expression, triggering floral induction [24]. Recent advances in *Arabidopsis* have refined the classical photoperiod models, specifically by distinguishing flowering-related and growth-related light signaling, and also incorporating dawn–dusk information. Plants were shown to measure daylength through photoreceptor-mediated timing, and also *via* metabolic daylength measurement mechanisms that coordinate with circadian outputs [40, 41]. Moreover, twilight length emerged as an additional dimension of the light environment influencing growth and flowering through core clock components [42, 43]. These updated frameworks provided an improved mechanistic reference for translating circadian-light principles from *Arabidopsis* to horticultural crops. Beyond describing daylength responses, recent work suggested that photoperiod regulation could be interpreted through two complementary mechanistic frameworks [42, 43]. One was external coincidence, in which circadian phase controlled the timing of light sensitivity so that light exposure coincided with specific clock states to activate downstream pathways (classically exemplified by CO/FT-type modules) [24]. The other emphasized daylength/energy-state measurement, where photosynthetic carbon gain and metabolic signals modulated clock outputs and growth programs, helping to separate timing cues from energy-dose effects [40, 41]. In addition, dusk/dawn transitions and twilight dynamics were increasingly recognized as informative signals that refined photoperiod perception beyond a simple ‘hours of light’ view, providing a clearer basis for interpreting diverse photoperiod phenotypes across horticultural crops.

In horticultural plants, the CO/FT regulatory module was widely conserved, and also exhibited species-specific variations. In *Fragaria* spp., wild diploid strawberries such as *F. vesca* were short-day plants that flowed in autumn after experiencing short photoperiods and low temperatures. Cultivated octoploid strawberries (*F. vesca × F. ananassa*) had been bred to display day-neutral or perpetual flowering phenotypes [44–46]. The CO-FT pathway remained functionally active in these species: FvCO promoted *FvFT1* expression under long days, and flowering was suppressed by the floral repressor FvTFL1 (TERMINAL FLOWER 1) of *F. vesca* [44, 45]. In long-day flowering mutants lacking functional TFL1 (H4 mutant), the inhibition of *FvCO* expression significantly delayed the flowering process, while its overexpression accelerated the flowering. This confirmed that in the absence of the inhibitory effect of TFL1, CO directly promotes the expression of *FT* and its downstream genes, such as *SOC1* and *AP1* [47]. In typical short-day genotypes, the CO/FT signaling pathway was transcriptionally active under long-day conditions. However, due to the inhibitory effect of TFL1, this pathway failed to trigger flowering in the higher plants [47, 48]. In perpetual-flowering cultivars, mutations reducing TFL1 activity released this repression, enabling FT-dependent flowering under long days. This revealed how modification of the CO/FT/TFL1 regulatory balance underlay the evolution of photoperiodic adaptation in strawberry [46, 49].

Chrysanthemum, another representative SDP, usually required short days to induce flowering. Recent researches demonstrated that manipulating light spectrum could modify this photoperiodic response of chrysanthemum. SharathKumar *et al*. found that extending long days with blue light promoted floral initiation in chrysanthemum. This suggested that light spectrum interacted with photoperiodic signaling [5]. On the molecular level, chrysanthemum flowering involved CO- and FT-like genes, such as *CsFTL3*, which acted as a key floral activator upregulated under short-day conditions [50]. Conversely, the antiflorigenic gene *AFT* (an *FT*-like repressor) was induced under long days, delaying flowering [51, 52]. Targeted nighttime illumination with specific wavelengths could modify the expression of these antagonistic genes, providing practical means to regulate flowering time in greenhouse production.

Cucumber was generally considered a day-neutral species, clear photoperiodic variation existed among ecological types. Low-latitude varieties, such as Xishuangbanna cucumber, exhibited distinct short-day flowering behavior, requiring day lengths of 8 to 11 hours for floral initiation [53, 54]. Transcriptomic analysis revealed that under long days, *CsFT* expression was suppressed, whereas under short days, *CsFT* and *CsCO* were upregulated synchronously. A transposable element insertion upstream of *CsFT* was found to correlate with short-day sensitivity by altering photoperiod-responsive promoter elements [55]. QTL localization revealed that a transposon insertion 15 kb upstream of the *CsFT* was strongly associated with short-day sensitivity, and this insertion affected the optical response element of the FT promoter [56]. In potato, photoperiodic tuberization was similarly controlled by *FT*-like genes. The FT-like StSP5G repressor activated by StCONSTANS-like1 under long days, and suppressed the tuberization signal StSP6A, thereby preventing tuber formation [57]. These examples illustrated that photoperiodic adaptation in horticultural plants may have resulted either from modifications in circadian output pathways (changes in CO/FT phase) or from *cis*-regulatory and functional divergence of downstream FT-like genes [36, 54].

### The regulation of growth and development by photoperiod of horticultural plant

Photoperiod determined the timing of flowering and further regulated vegetative growth, morphogenesis, and metabolic activity through circadian signaling. In horticultural plants, this regulation exhibited remarkable species specificity and plasticity. In *Brassica rapa* var. *chinensis* (pak choi), extending the light duration from 12 to 21 hours significantly enhanced growth rate and energy-use efficiency [58]. Similarly, in greenhouse-grown tomato, extending the photoperiod to 20 h increased photosynthetic rate, chlorophyll content, and plant dry weight, while mineral nutrient levels remained largely unchanged [59]. These results indicated that moderate photoperiod extension under artificial lighting can substantially improve the yield and quality of horticultural crops.

The optimal photoperiodic window varied widely among species. In celery (*Apium graveolens*), comparison among 8 h/16 h, 12 h/12 h, and 16 h/8 h light/dark cycles revealed that a neutral 12-hour photoperiod produced the most vigorous growth and the highest nutritional quality, including soluble sugars, vitamin C, and total phenolics [32]. Excessively long or short photoperiods could lead to photodamage, metabolic imbalance, or photoinhibition, underscoring the need for precise photoperiod optimization based on species and developmental stage. When plants were subjected to skeleton photoperiods (6 L/6D, 6-h light/6-h dark), both tomato and tea plant (*C. sinensis*) exhibited phase delays and reduced oscillation amplitude of clock genes, indicating that nonstandard light–dark cycles could disrupt circadian synchronization [33, 60].

In addition to directly influencing photosynthesis of higher plants, photoperiod also regulated the internal developmental program through the signal transduction network. Plants perceived changes in photoperiod through multiple photoreceptors, including phytochromes, cryptochromes, and phototropins, which relayed signals to the circadian gene network, thereby regulating downstream developmental regulators [61]. Recent literature reported on the differences and characteristics between absolute photoperiod and photosynthetic photoperiod. The absolute photoperiod primarily affected cell division, branching, and hormonal balance, while the photosynthetic photoperiod governed fruit and seed development through carbon assimilation and energy allocation [62]. Extending the absolute photoperiod promoted shoot branching and fruit number, whereas extending the photosynthetic photoperiod enhanced dry matter accumulation in fruits [63].

In woody horticultural species, photoperiod often interacted with temperature to regulate dormancy and bud break. In deciduous fruit trees, such as apple and pear, shortened day length in autumn triggered bud dormancy, a process believed to involve both circadian and light-signaling pathways [2]. The results in *Arabidopsis* also showed that the biological clock regulated the effects of photoperiod on seed dormancy and leaf senescence. Shorter periods of daylight promoted dormancy and delayed the aging process [64]. Although direct evidence remained limited in woody perennials, the similar mechanisms were likely conserved with model plant. In horticultural production, the night-break technique was widely used to manipulate dormancy and flowering in short-day species. A brief illumination during the middle of a long night interrupts the plant's measurement of continuous darkness, preventing the induction of flowering or dormancy [65, 66]. In chrysanthemum cultivation, night-time red-light exposure effectively suppressed floral differentiation and maintains vegetative growth [67]. These classic observations revealed that the circadian clock defines specific ‘windows of light sensitivity’ during the night, when red light was particularly effective due to the clock-controlled activation of light-responsive factors.

Overall, photoperiod acted as a key environmental signal linking external day length with internal developmental programs *via* the circadian clock (Table 1). Through long-term domestication and breeding, horticultural crops had evolved diverse strategies to adapt to photoperiodic cues. For instance, the CO/FT–TFL1 interaction in strawberry that released floral repression, to spectral regulation of flowering plasticity in chrysanthemum, and FT-promoter variations conferring photoperiodic diversity in cucumber and other horticultural crops. Photoperiod had a significant impact on flowering, and it also positively influenced vegetative growth, carbon allocation, dormancy, and senescence. This highlights the central role of the biological clock in coordinating the physiological and metabolic rhythms of horticultural plants.

**Table 1:** Effects of photoperiod regulation in growth and flowering of horticultural plants

Mechanism/Key factors	Representative species	Main effect	Application	Reference
CO-FT module	Strawberry [44–49], chrysanthemum [5, 41–43], cucumber [53–56], potato [57]	The coincidence of CO and light determines the activation of FT, inducing or inhibiting flowering	Regulate flowering through day length control or night-break	[5, 44–57]
Growth/reproductive distribution output	Cabbage [58], tomato [59], celery [32], fruit tree [2, 65, 66], tea plant [53], chrysanthemum [67]	The length of the day affects vegetative growth and reproductive conversion, as well as yield/quality	Adjust the duration of sunlight according to the growth stage to optimize the yield	[2, 32, 53, 58, 59, 65–67]

## Circadian rhythms and light quality: mechanisms of spectral response of horticultural plants

Higher plants were capable of keeping track of day and night duration, and were equally perceptive to light quality (spectral composition). In natural environments, the spectrum of sunlight changed continuously from dawn to dusk: the red/far-red (R/FR) ratio decreased and the blue light intensity varied, and these changes were perceived by higher plants and were used to adjust their internal circadian rhythms [68, 69]. Multiple classes of photoreceptors detected specific wavelength ranges: phytochromes (PHYs) perceived red and far-red light; cryptochromes (CRYs) and phototropins (PHOTs) responded to blue and UV-A light; and UVR8 detected UV-B radiation [12, 13]. Light influenced the circadian clock through both direct and indirect routes. Direct inputs included photoreceptor-dependent regulation of core clock components and clock-associated hubs. Indirect inputs were mediated by downstream light-signaling networks that reshaped clock outputs and feedback. In particular, PHYTOCHROME-INTERACTING FACTORS (PIFs) acted as key intermediates linking phytochrome signaling to circadian-gated transcriptional programs. Recent models emphasized these multi-layer connections beyond classical photoreceptor-only descriptions [70]. The work from the Imaizumi group showed that PHYTOCHROME-INTERACTING FACTOR 7 contributes to FT regulation. Under natural long-day conditions, they also demonstrated that the phyA-mediated high-irradiance response and the external coincidence mechanism contribute to morning FT induction [71].

The expression and activity of circadian clock genes were often regulated by these photoreceptors, enabling spectral shifts to influence the phase and period of the biological clock. The R/FR light signal formed a key component of light-quality responses and circadian regulation in woodland strawberry and rose. Plants perceived changes in the R/FR ratio through PHYs, which in turn modulated morphogenesis, circadian phase, and metabolic activity [72, 73]. The active (Pfr) and inactive (Pr) forms of PHYs could interconvert under red and far-red light, allowing plants to sense both daylength and neighbor-induced shading [74].

R/FR signaling profoundly affected photoperiodic rhythms and physiological metabolism. In tomato, a low R/FR ratio promoted the expression of genes associated with elongation growth, while repressing genes related to photosynthesis and flavonoid biosynthesis. Consequently, anthocyanin and chlorophyll contents, as well as photosynthetic capacity, decreased in tomato [75]. Under continuous light, alternating red and blue illumination could mitigate photoperiodic stress, maintain rhythmic stability, and prevent photoinhibition in tomato [76]. In cold tolerance, phyA enhanced cold tolerance by activating ABA-dependent jasmonate signaling and the downstream CBF cold response pathway, whereas phyB exerted an opposing effect in tomato [77]**.** These findings indicated that the R/FR ratio modulated photosynthetic rhythms and energy allocation through PHY circadian clock interactions. In ornamental species, R/FR signaling primarily regulated floral induction and plant architecture. Under a light regime consisting of 11 hours of far-red irradiation followed by 4 hours of blue irradiation, flowering in chrysanthemum was inhibited, while vegetative growth was promoted. This was associated with upregulation of *CmPHYA* expression and suppression of *CmFTL3* expression [78].

In contrast, supplemental red light with a high R/FR ratio promoted PHY activation and flower bud formation [79]. In *Matthiola incana* (stock), a low R/FR ratio or nocturnal far-red irradiation accelerated flowering and internode elongation, whereas a high R/FR ratio delayed flowering and resulted in dwarf phenotypes [80]. The R/FR ratio also influenced photosynthetic metabolism and fruit quality. A reduced R/FR ratio under salt stress enhanced the accumulation of carbohydrates, phenolics, and amino acids in tomato, thereby improving antioxidant capacity and fruit quality [81]. In grapevine, red and blue light exposure around fruit clusters promoted the accumulation of anthocyanins, flavonoids, and other phenolic compounds, enhancing the nutritional quality of the berry skin [82]. These results collectively indicated that during fruit development, the R/FR signaling regulated reproductive transition and profoundly affected the accumulation of photosynthates and secondary metabolites.

Blue light, perceived mainly by CRYs, modulated both the circadian clock and photoperiodic responses. As a major spectral component in morning and midday sunlight, blue light suppressed the accumulation of core circadian repressors, such as TOC1, thereby accelerating the clock pace under steady illumination [83]. In horticultural crops, the functions of the PHY and CRY families showed significant diversification. In tomato, manipulation of cryptochrome gene expression (*CRY1A*, *CRY2*) has been shown to substantially alter diurnal and circadian transcript oscillations, indicating functional impacts of specific *CRY* paralogs of *Arabidopsis* [84, 85]. In strawberry, FvCRY1 and FvCRY2 were functionally characterized to directly regulate anthocyanin biosynthesis and sugar metabolism under blue light, revealing species-specific signaling pathways involving cryptochromes [86]. Higher plants exhibited shortened periods and reduced rhythmic amplitudes under monochromatic blue light, owing to the absence of red-light input [87]. In *Arabidopsis*, blue light specifically modulated the activity of certain clock components and outputs. Hughes *et al.* reported that the members of the REVEILLE family showed distinct contributions under blue versus red light: RVE8 exerted stronger activation of downstream targets under blue light, while RVE4 and RVE6 were more active under red light [88]. This light-quality-dependent functional differentiation enabled plants to fine-tune their circadian networks in response to spectral changes throughout the day [88]. Morning light, enriched in blue wavelengths, likely enhanced the expression peaks of dawn genes *via* CRY-mediated pathways, whereas the red light at dusk promoted the expression of evening genes through PHY signaling, ensuring that the oscillator remained synchronized with the actual day–night cycle.

In horticultural crops, the role of blue light in photoperiodic regulation had also attracted increasing attention. As shown in chrysanthemum, nighttime extension with blue light exerted an effect opposite to that of red light [5]. In some long-day horticultural species, blue night-time supplementation was less effective than red light and might not inhibit flowering [89]. Kong *et al.* demonstrated that pure blue light (450 nm) promoted hypocotyl and petiole elongation in horticultural species, such as arugula, cabbage, and kale, mimicking a ‘shade-like’ response [90]. Blue light also affected leaf expansion and plant compactness. Adjusting the red-to-blue light ratio had therefore become a major approach in LED-based greenhouse lighting. Generally, red light promoted elongation and was high-energy utilization efficiency, whereas blue light suppressed excessive elongation, enhanced compact morphology, and influences flowering time. Thus, optimizing red–blue combinations enabled control over growth rate and the shape of the plants. For example, in cuttings of azalea and chrysanthemum, increasing the blue component promoted stronger seedlings and earlier flowering. In leafy vegetables, enhanced blue light improved nutritional quality, and maybe reduced leaf area. A balance needs to be struck between these two factors [91].

The influence of multi-spectral combinations on the circadian clock represented an emerging research frontier. In tea plants, the photosynthetic cycles under red, blue, purple, and white light were compared, and it was found that there were differences in the diurnal oscillation of net photosynthesis under different light qualities treatments. Under purple and white light, the amplitude of photosynthetic rhythms was greater, while it was reduced under monochromatic red or blue light, suggesting that multiple spectral cues acted synergistically to maintain robust circadian oscillations in tea plant [87]. Lanoue *et al.* [76]*.* applied a dynamic light regime, red light during the day and low-intensity blue light at night in greenhouse-grown tomato. This successfully avoided photoperiodic damage under continuous illumination and achieved a yield increase comparable to conventional photoperiod treatments. The result suggested that nocturnal blue light served as a weak-light signal, exerting minimal disruption to circadian timing while allowing partial maintenance of nighttime physiological processes, such as respiration and cellular repair [76]. The dynamic spectral strategy had pointed out the future development direction for controllable agricultural environments by using spectral modulation to synchronize the circadian clock and optimize the growth performance of crops [92].

After discussing the effects of photoperiod and light intensity on flowering and growth, the distinctions between photoperiod, daily light integral (DLI), and light intensity were further elaborated. Photoperiod referred to the duration of light exposure, which served as a temporal signal that regulated circadian rhythms and seasonal processes, such as flowering and dormancy. In contrast, daily light integral quantified the total amount of light received over a 24-hour period, combining both light intensity and exposure duration. The DLI signal influenced photosynthesis and growth. Light intensity was the instantaneous amount of light received by the plant, which directly impacted photosynthetic activity and metabolic processes.

In summary, the effects of different wavelengths on circadian and developmental processes in horticultural plants were strongly spectrum dependent (Table 2). Red and far-red light, perceived through PHYs, convey information about daylength and R/FR ratios, thereby regulating flowering and morphogenesis. Blue light, perceived *via* CRYs and related photoreceptors, influenced circadian periodicity and photosynthetic metabolism while also affecting morphology and flowering. In many long-day species, far-red enrichment (a reduced R/FR ratio) could accelerate flowering. This effect was highly context-dependent and typically depended on the spectral background and timing, end of day far-red or far-red extension under long-day conditions. In some cases, red-light pre-exposure or sufficient red light was required for a consistent promotive response [93]. As our understanding of the interactions between light quality signals and the circadian clock deepens, it will be possible to design more refined lighting schemes to enhance greenhouse productivity and horticultural crop quality.

**Table 2:** Effects and applications of light quality on growth and rhythmic regulation of horticultural plants

Mechanism/Key factors	Representative species	Main effect	Application suggestions	Reference
Red/far-red (R/FR) ratio	Tomato [75–77, 81], chrysanthemum [78, 79], violet [80], grape [82]	The shading response, elongation and flowering period are regulated by PHYs (low R/FR often promotes elongation)	Adjust R/FR or provide supplementary lighting at night to control plant type and flower bud differentiation	[75–82]

Blue light (cryptochromes/phototropins)	Chrysanthemum [5, 89, 91], arugula [90], leaf vegetables [91], tea plant [87]	It affects stomata, chlorophyll, rhythm phase and amplitude, and improves quality	A certain proportion of blue light is retained in LED to stabilize the rhythm and improve the quality	[5, 87, 89–91]

Spectral combinations (red + blue + far red)	Strawberry [67], tea plant [76]	The compound band is superior to the single band and can precisely regulate morphology and metabolism.	Dynamic spectroscopy is adopted according to the growth stage (with differentiated light distribution during the induction period and growth period).	[67, 76]

## Regulatory effects of light intensity on plant circadian rhythms and physiological metabolism of horticultural plants

Light intensity determined the energy input for photosynthesis and affected the characteristics of circadian rhythms in higher plants [94]. In natural environments, variations in light intensity caused by changing weather or canopy shading could lead to fluctuations in photosynthetic and transport rhythms in leaves [95]. In artificial environments, manipulating light intensity and duration was also a crucial factor influencing crop rhythms [96]. Generally, high light intensity enhanced the amplitude of circadian oscillations, whereas weak light intensity or prolonged darkness reduced rhythmic amplitude and lengthened the period. Plants required sufficient light cues to resynchronize their internal clock each day. Otherwise, under weak light intensity or continuous darkness, the oscillation became imprecise or even disappeared [97].

Among vegetable crops, tomato was an important species for studying the relationship between light intensity and circadian rhythms. Excessive light intensity or continuous illumination (>500 μmol∙m^−2^∙s^−1^) disrupted rhythmicity, caused chlorophyll degradation, and induced photoperiodic injury [98]. Under supplemental lighting regimes, alternating strong–weak LED illumination could restore rhythmic oscillations, allowing tomatoes to maintain stable growth under long-day or continuous light conditions [76]. Under low-light stress, shade-tolerant cucumber cultivars exhibited stronger adaptability by maintaining higher photosynthetic efficiency and upregulating genes associated with photosystem stability [99].

In leafy vegetables, light intensity played a decisive role in rhythm driven photosynthetic efficiency and nutritional quality. In lettuce (*Lactuca sativa*), low light intensity (150 μmol∙m^−2^∙s^−1^) significantly weakened photosynthetic rhythmicity and disrupted carbon metabolism, while high light intensity (300–400 μmol∙m^−2^∙s^−1^) restored the 24-hour oscillation of *LsPRR9* and *LsGI* and enhanced biomass accumulation [100]. In spinach (*Spinacia oleracea*), low light intensity (200 μmol·m^−2^·s^−1^) significantly reduced biomass accumulation and redistributed assimilates to leaves, whereas higher light intensity (800 μmol∙m^−2^∙s^−1^) favored carbon assimilation and metabolite synthesis [101].

In ornamental plants, the interplay between light intensity and circadian rhythms primarily manifested in photoperiodic morphogenesis and floral induction. In lily (*Lilium spp.*), different developmental stages (leaf expansion, bud formation, flowering) showed distinct physiological responses to light intensity. The increase in light intensity enhanced the photosynthetic capacity and the accumulation of dry matter, especially after the bud stage. Intense light significantly promoted the differentiation of flowers and the development of organs, thereby increasing the number and quality of flowers. In contrast, moderate shading during early growth promoted leaf expansion and stem elongation, indicating a stage-specific regulation of morphogenesis and carbon allocation by light intensity [102]. Chrysanthemum, a typical short-day plant, exhibited a tight coupling between photoperiodic induction and circadian rhythm. Zhang *et al.* found that several photoperiod-related genes (*CmLHY*, *CmGI*, and *CmFTL3*) displayed characteristic oscillatory patterns under varying light intensities. Under short-day and low-light conditions, the expression peaks of *CmLHY* and *CmGI* were delayed with reduced amplitudes, while *CmFTL3* transcription was induced, accelerating floral induction and flowering [103]. These results indicated that changes in light intensity precisely controlled the flowering time of short-day plants by regulating the phase and amplitude of the core circadian clock genes. Additionally, under lower light intensity (~16.8 μmol∙m^−2^∙s^−1^), different ornamentals exhibited species-specific differences. *Petunia hybrida* and *Salvia splendens* flower earlier, while *Impatiens walleriana* and *Zinnia elegans* maintained longer flowering durations (>3 weeks), indicating stronger low-light tolerance. When light intensity falls below 8.4 μmol∙m^−2^∙s^−1^, even shade-tolerant species lose normal coloration and ornamental value [104].

In woody horticultural crops, the coordination between light intensity and rhythmic regulation was equally critical. In grape (*Vitis vinifera*), higher light intensity promoted callus formation, enhanced enzyme activities, and upregulated regulatory genes, such as *PAL*, *POD*, and *CHS*, thereby accelerating tissue differentiation [105]. Beyond intensity-driven responses, researches in woody perennials showed that seasonal photoperiod cues were integrated with endogenous rhythmic programs to regulate growth cessation, dormancy progression, and subsequent bud break. In Populus, photoperiod sensing modules involving photoreceptors (e.g. PHYB) and clock-linked regulators converged on FT-like targets that coordinated the annual growth cycle [106]. In Prunus species, DORMANCY-ASSOCIATED MADS-box (DAM) factors were repeatedly implicated as key regulators of dormancy cycling, providing a mechanistic entry point for linking seasonal cues to developmental transitions [107].

Recent advances in spectrum-tunable and dynamic LED technologies have enabled real-time control of light quality, intensity, and timing, offering opportunities to optimize circadian alignment and photosynthetic efficiency in horticultural crop production system. For instance, tunable LED ‘light recipes’ that synchronize with plant circadian rhythms have been proven to improve biomass accumulation, metabolite balance, and energy efficiency [108, 109]. Different light spectra determined photosynthetic efficiency and influence plant perception and adaptation to light intensity through the coupling of photoreceptor–circadian–metabolic networks [110, 111]. Simply adjusting the light intensity or spectrum often could not achieve the best physiological effect. However, dynamically combining the intensity and spectrum of light could enable the biological rhythm to be coordinated with the utilization of light energy. For example, varying blue-light intensities (0–40 μmol∙m^−2^∙s^−1^) or nighttime blue-light interruption (NI-B) significantly influenced chrysanthemum flowering and carbon assimilation. Blue light intensity at 30 μmol∙m^−2^∙s^−1^ most effectively promoted flowering, chlorophyll accumulation, and sugar content, whereas 40 μmol∙m^−2^∙s^−1^, despite enhancing photosynthesis, disrupted the balance of floral induction genes, hindering flowering [112]. Blue light regulated chrysanthemum flowering *via* both photosynthetic products and photoreceptor-mediated pathways, acting as a key factor in light intensity–dependent photoperiodic flowering [112]. In strawberry (*Fragaria × ananassa*), blue-light supplementation or nighttime blue-light intervention (NI-BL) markedly regulated photoperiodic flowering and runner formation. Blue light intensity (20 μmol∙m^−2^∙s^−1^) effectively promoted flower bud initiation by modulating circadian genes, such as *FaFT1* (upregulated) and *FaTFL1* (downregulated), resetting photoperiodic responses [113].

In summary, light intensity and light pattern affected the amplitude and period of the circadian rhythm, thereby influencing photosynthesis, metabolism and growth. In horticultural crops production, balancing light-use efficiency and circadian stability was essential [3]. Although high light intensity and extended photoperiods could increase horticultural crop yield, exceeding the tolerance range of species’ circadian rhythmic systems can be detrimental. The concept of ‘intelligent lighting’, such as nighttime dim light, spectral mixing, and gradual transitions in brightness, represented a current research frontier in controlled-environment horticulture. The core idea was to provide sufficient light energy without disturbing intrinsic circadian programming-achieving high productivity without desynchronization (Table 3).

**Table 3:** Effects of light intensity on growth and corresponding regulation strategies of horticultural plants

Mechanism/Key factors	Representative species	Main effect	Application suggestions	Reference
High light	Tomato [60, 98], lettuce [100], spinach [101], lily [102], grape [105]	The daily light accumulation determines the dry matter accumulation, flowering quality and leaf development.	Adjust R/FR or provide supplementary lighting at night to control plant type and flower bud differentiation	[60, 98, 100–102, 105]

Low light	Tomato [98], cucumbers [99], spinach [101], chrysanthemums [103], tea plant [52], flowers [104]	It affects stomata, chlorophyll, rhythm phase and amplitude, and improves quality	Use supplemental lighting to maintain the minimum DLI for rhythm stabilization during low-light seasons	[52, 98, 99, 101, 103, 104]

## Mathematical model of the biological clock of horticultural plants

**Figure 3:**
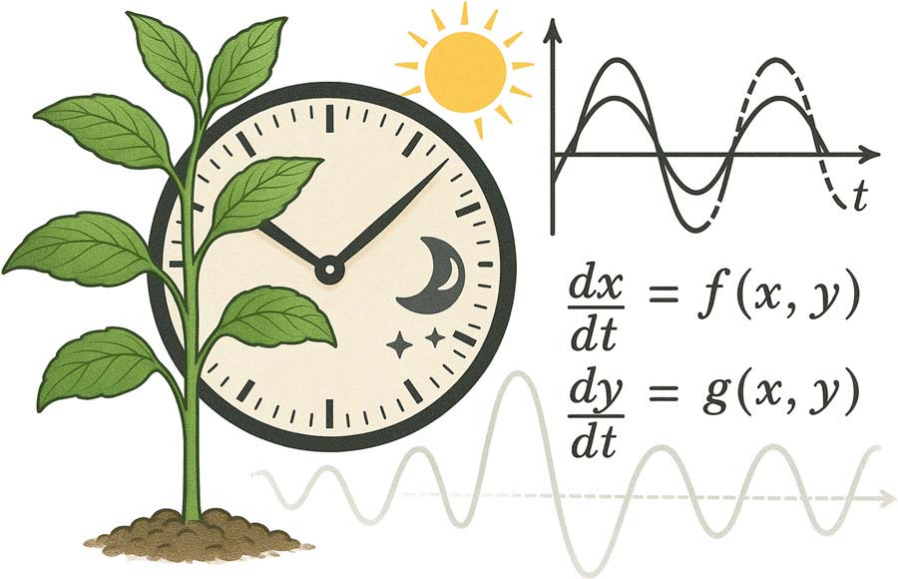
Integration of plant circadian rhythms with mathematical modeling. The clock was represented as a dynamical system in which state variables *x* and *y* denoted core clock states (representative gene-module activities or phase/amplitude), and functions *f* (·) and *g* (·) captured feedback regulation and environmental forcing. In practical applications, light and temperature entered the model as time-dependent inputs (photoperiod, PPFD/DLI, spectral channels, and thermal cycles), enabling prediction of phase shifting/entrainment and downstream trait dynamics under programmable lighting regimes.

Mathematical modeling of the circadian clock in horticultural plants aimed to establish a quantitative framework to elucidate how the transcription-translation feedback loops (TTFL) formed a complex oscillatory network. This feedback loop maintained a stable 24-hour rhythm under both daily and seasonal light conditions, and accurately transmitted time information to the processes of photosynthesis, metabolism, and development (Fig. 3) [114, 115]. Most established models were based on *Arabidopsis* and abstracted the CCA1/LHY—PRR—Evening Complex feedback loops into a set of nonlinear ordinary differential equations. By parameterizing transcription, translation, protein degradation rates, and inhibitory/activating interactions, these mathematical models reproduced the phase, amplitude, and period characteristics of key circadian genes under continuous or cyclic light conditions [115, 116]. Huang and her colleagues incorporated photoreceptor-mediated light input functions (PHYs and CRYs), as well as external drivers such as temperature and sugar signaling, constructing an integrated ‘core oscillator + multi-input/multi-output’ mathematical modeling framework to explain entrainment and resetting of the clock by light quality (red/far-red, blue), light intensity, and temperature [117]. The types of mathematical models provided a structural blueprint for extending circadian modeling to various horticultural crops.

In horticultural plants, an important direction in mathematical modeling had been the coupling of the core circadian oscillator with functional processes, such as photosynthesis, stomatal conductance, and carbon assimilation. The mathematical models enabled quantitative analysis of how different light environments integrated ‘rhythm–photosynthesis–growth’ regulation. In tomato, for example, Huang *et al.* developed a ‘clock–photosynthesis’ coupled model that described the circadian modulation of key parameters associated with photosynthetic electron transport and carbon assimilation, and simulated the dynamic changes in net photosynthetic rate under varying light intensities. Their results demonstrated that the circadian period was markedly lengthened under low-light conditions (<200 μmol·m^−2^·s^−1^), and the peaks of photosynthesis and carbon balance were delayed, thereby reducing light-use efficiency [60]. In tea plant, Hu *et al.* introduced a ‘skeleton photoperiod (6L/6D)’ as an intermittent light-input signal into a TTFL-based mathematical model and coupled it with a photosynthesis–stomatal conductance module. They found that discontinuous illumination disrupted phase relationships among multiple clock outputs, causing misalignment between photosynthetic and stomatal rhythms and significantly reducing light-use efficiency [32]. In leafy vegetables of celery, well-designed skeleton photoperiods enhanced the synchronization between circadian regulation, photosynthesis, and metabolic processes, leading to improved photosynthetic efficiency and yield [118].

The multi-segment light intensity model and the red-blue alternating LED continuous illumination models have explicitly incorporated parameters, such as effective light exposure windows and circadian sensitivity periods into crop growth models. These frameworks enabled the assessment of leaf photosynthetic capacity, biomass accumulation, and photoinhibition risks under different daily light integrals and combinations of spectrum and intensity. The frameworks provided quantitative guidance for optimizing light recipes in the controlled-environment cultivation of crops like tomato, lettuce, and other horticultural crops [119, 120]. For controlled-environment horticulture production, modeling constructed a quantitative bridge from clock mechanisms to management decisions. The method based on synchronization and phase response curves could predict the phase changes and recovery times under changes in the light cycle. Mathematical models or hybrid models evaluated how photosynthetic photon flux density, daily light integral, and spectral schedules influenced oscillator robustness and downstream outputs, such as photosynthesis, metabolite accumulation, and flowering. These frameworks supported rhythm-aligned lighting optimization and helped prioritize clock-associated targets for breeding of horticultural crops or engineering design of efficient production for horticultural crops.

## Future perspectives

In recent years, circadian rhythms research in horticultural plants had advanced substantially, yet key translational challenges remained. The underlying mechanisms of the biological clocks in many horticultural crops were still not well understood. Across the species discussed here (e.g. strawberry, chrysanthemum, tomato, cucumber, tea plant, and grape), a recurring principle was time-of-day dependent ‘gating’, whereby identical light cues elicited distinct outputs depending on internal circadian phase. Interspecific variation mainly reflected horticultural crops and target traits. Photoperiod-induced flowering control was most prominent in ornamentals, while light dose and source–sink dynamics dominated in fruit/vegetable crops, and secondary metabolism rhythms shaped quality in tea plants. The seasonal dormancy programs were integrated with photoperiodic timing in woody perennial plants. Therefore, by comprehensively analyzing the dimensions of light (photoperiod, spectrum, intensity) and the clock-output module of the biological clock, it may be possible to more effectively promote consistency across higher plant species, including model plants and horticultural plants. This perspective aligned with the concept of chrono culture, which proposed improving performance and resource-use efficiency by optimizing the timing of agronomic interventions and leveraging genetic variation in circadian regulation [42, 121].

### Diversity of circadian clocks and adaptation of horticultural crops

The circadian networks of different horticultural species might have possessed unique components or regulatory modes, providing a genetic basis for crop adaptation to diverse environments [25]. For instance, the opposite roles of the CO/FT pathway in short-day and long-day plants, as well as clock regulation associated with dormancy in perennial species, indicated that circadian mechanisms varied according to species and life history [24]. Future research could have focused on identifying the circadian clock genes in plants and mapping out the rhythmic networks in a wider range of non-model horticultural plants. Some horticultural species that have received relatively little research and have complex genetics, such as the tea plant (*C. sinensis*) and orchids (*Cymbidium* ssp.), have also recently become subjects of studies related to circadian rhythms. In tea plants, a ‘skeleton photoperiod’ treatment that mimics dappled light under mountain canopies has revealed that intermittent illumination significantly alters photosynthetic rhythms and stomatal behavior, indicating that the circadian clock exhibits complex responses to discontinuous light cues of tea plants [32]. The results expanded the taxonomic scope of circadian research and test the applicability of existing models across species. In the future, greater attention could be devoted to perennial fruit trees (grapevine, apple, orange) and long-lived ornamental plants to fill the current knowledge gap dominated by annual herbaceous models and to better understand annual rhythm cycles and seasonal adaptation in perennials.

### Circadian regulation quality traits of horticultural crops

The circadian clock affected flowering time, growth rate, quality and resistance traits in horticultural crops. Clock-controlled genes regulated the diurnal accumulation of aroma compounds, the rhythmic metabolism of sugars and organic acids in fruits, such as tomato, and the dynamic levels of nutritional substances in vegetables [1, 122]. Improving horticultural crops quality might therefore require consideration of circadian timing. Aromatic flowers (rose, *Nicotiana alata*) emitted fragrance at specific times of the day, a process associated with circadian regulation of volatile biosynthetic enzyme expression. Similarly, the rhythmic oscillation of sugar accumulation and organic acid degradation in fruits suggested that harvest timing could influence flavor quality.

Future research can explore the influence of circadian rhythm regulation on the secondary metabolism of horticultural plants. It could also investigate the possibility of optimizing the accumulation of secondary metabolites by regulating the photoperiods or mining rhythm regulators, thereby enhancing the flavor and nutritional value of horticultural crops. The research on the circadian rhythm regulation of quality traits could be adjusted according to the type of horticultural crop. In tea plants and other beverage/medicinal crops, priorities include clock-linked control of polyphenols/catechins and aroma-related pathways. In tomato, particular attention could be paid to indicators related to flavor and nutrition, such as the balance of sugar and acid, and the accumulation of pigments at different stages of ripeness. In ornamentals, key targets are time-of-day dependent floral scent emission and pigmentation. In leafy vegetables, attention could focus on nutritional quality (e.g. vitamin C, nitrate/antioxidants) and postharvest rhythmic persistence. Across categories, standardized diel sampling and linking core quality metrics to clock outputs also essential.

### Circadian rhythms and stress resistance in horticultural crops

The networks governing plant stress responses were closely intertwined with the circadian clock, resulting in ‘time-of-day–specific’ resistance patterns. For instance, plant defense against powdery mildew and herbivorous insects fluctuated throughout the day due to circadian regulation of key defense hormones, such as jasmonic acid (JA), and secondary metabolites [123, 124]. In horticultural crops, many pests and pathogens also displayed diel activity cycles. Aligning the horticultural crop's defense rhythms with pest activity peaks could enhance control efficiency, offering a promising strategy for integrated pest management in vegetable production [125]. The research showed that vegetables, such as cabbage, have been harvested, can still maintain their biological clock rhythms in a simulated day-night alternation environment. This significantly enhances their ability to resist pests and preserves their nutritional content. The finding suggested that postharvest management can also harness circadian principles to ‘train’ produce for improved quality [126]. The approach opens new possibilities for horticultural storage and preservation technologies, such as through controlled light exposure to maintain antioxidant capacity and enzyme activity during storage and beyond the shelf life.

### Photoperiodism under environmental change in horticultural crops

Climate change has led to mismatched between seasonal photoperiods and temperature patterns, as seen in regions where warmer winters disrupt the synchronization between vernalization cues and daylength signals [1]. Simulating the changes in future light cycles and temperature conditions can provide valuable reference information for horticultural crops breeding and production planning. For instance, shortened winters or extreme daylength fluctuations (intensified polar day/night cycles at high latitudes) may adversely affect flowering and fruiting. In controlled environment production, similar time conflicts may also occur. In the environment, prolonged or excessive lighting can disrupt the phase and amplitude of the circadian rhythm, and the extent of these effects varies depending on the species and variety of horticultural crops. Circadian-based breeding modifying key clock genes to fine-tune circadian periods could enhance horticultural crops adaptability to atypical photoperiods. Identifying or creating alleles with broader photoperiod adaptability may enable horticultural crops to better withstand climatic uncertainties.

### Interdisciplinary research and design of horticultural crops

Circadian biology is increasingly integrating with synthetic biology and computational modeling to achieve quantitative understanding and rational reconstruction of complex rhythmic systems [32, 127]. Mathematical models can simulate the impact of different light regimens on growth and assist in optimizing greenhouse environmental control parameters of horticultural crops [60]. Gene-editing technologies enable precise modification of clock-associated genes in horticultural crops. These advancements have facilitated the development of the concept of ‘ horticultural crop design based on circadian rhythms’, which involves genetically modifying crop varieties to enable them to possess the biological clock parameters that are compatible with specific ecological or cultivation conditions. For instance, horticultural crops in high-latitude regions may require longer periods and more significant seasonal variations to fully utilize the longer daylight hours during summer, while indoor vertical farming systems need to accommodate continuous lighting while maintaining regular horticultural crops. In the future, the characteristics of circadian rhythms are expected to become an equally important breeding target as yield and quality characteristics in horticultural crops.
